# Editorial: Nutrition in vulnerable groups

**DOI:** 10.3389/fnut.2024.1448870

**Published:** 2024-09-06

**Authors:** Enbo Ma, Yukiko Wagatsuma

**Affiliations:** ^1^Health Promotion Center, Fukushima Medical University, Fukushima, Japan; ^2^Department of Epidemiology, Fukushima Medical University School of Medicine, Fukushima, Japan; ^3^Department of Clinical Trials and Clinical Epidemiology, University of Tsukuba Faculty of Medicine, Ibaraki, Japan

**Keywords:** food security, life stage, COVID-19, vulnerable people, malnutrition, strategy and policy

To maintain good health and prevent disease, essential and adequate nutrition is warranted, particularly among vulnerable people at different life stages. Uncertain physical, social, cultural, and economic situations limit access to nutrition and influence the health of those living in conditions of poverty, unemployment, lack of schooling, addiction, and ethnic minorities (1). Furthermore, individuals or families facing financial insecurity are restricted in their ability to acquire knowledge regarding proper nutrition (2). Struggling to adhere to healthy diets and often selecting cheaper and unhealthy options lead to excessive consumption of inappropriate supplements. Malnutrition affects individual health and productivity, inversely impacting a country's economic growth (3).

This Research Topic features the role of nutrition science in dietary behavior, food quality, and nutrients, not only in their associations with health outcomes but also for education, knowledge, and practice of vulnerable populations. It also published 14 articles, covering the most relevant topics in both developing and developed countries, and providing novel findings and recommendations on healthy nutrition practices.

First, Shreffler et al. reported in a cohort study of pregnant women with low-income mothers in a south-central US state that periconception food insecurity was positively associated with parenting stress at 2 months postpartum, suggesting that these negative impacts on parental wellbeing and parent-child relationship early in the infant's life. In a review of published data between 2011 and 2023, Assaf et al. demonstrated that exclusive breastfeeding was 24.4% in the Gaza Strip compared to a national rate of 39.9% in 2020 in Palestine. The authors appeal to governments for strategies that prioritize nutritional interventions to attain sustainable development goals. It should be noted that on the date of writing the editorial, the Israel and Palestine War was ongoing in Gaza, and homeless people were suffering from poor nutrition.

This Research Topic contains eight articles on children. Assaf et al. also reported that 7.3, 14.5, and 15.7% of schoolchildren in the West Bank of Palestine were underweight, overweight, and obese, respectively. Similarly, Vastrad et al. observed in a study of 2,700 school-age children in India that the prevalence of stunting and severe stunting was 19.5 and 7.6%, respectively, whereas Kalinda et al. observed rates of 19.2 and 12.2%, respectively, in 2,788 children under 5 years old in Rwanda. Both studies revealed that socio-demographic and environmental factors were significant determinants of childhood stunting. To manage 402 outpatients aged < 5 years with severe acute malnutrition in East Ethiopia, Yadeta et al. reported a 0.7% death rate and an 89.6% cure rate, which remain below the universal target for curing children. Enhanced health education packages and community engagement are necessitated to improve early recovery. Meanwhile, through dietary patterns identified in a survey of 510 adolescents, Gedamu et al. reported that 22.5% of overweight and 6% of obese adolescents were in East Ethiopia, highlighting that overnutrition is associated with dietary consumption patterns, eating behaviors, wealth status, literacy, and level of physical activity among adolescents. In a cross-sectional study of African American adolescents, Ardakani et al. confirmed that developing culture-based nutritional education programs is crucial among parents and youth.

Malnutrition continues to exist among adult ethnic groups and has increased in Thailand since 2014. Pechdin and Bunditsakulchai investigated the causes of malnutrition risk among 981 people with income insecurity using telephone-based survey data from the United Nations Food and Agriculture Organization. Among women aged 30–44 years, 11.5% did not have access to adequate nutrition. The authors propose the development of mid- and long-term programs and vocational training opportunities for enhancing employment and income stability. In a 3,491-household survey, Hernández-Vásquez et al. first reported that 39% of Venezuelan immigrant households in Peru experienced moderate-to-severe food insecurity, as established by the Food Insecurity Experience Scale. In a study of 497 prisoners in Ethiopia, Wondimu et al. reported that 20% were undernourished and that financial support, duration of imprisonment, dietary diversity, and depression were associated with undernourishment. The aforementioned study of Assaf et al. also indicated that 57.8% of overweight and 26.8% of obese adults were in Palestine, emphasizing the double burden of malnutrition associated with social determinants.

Furthermore, malnutrition among older adults is a highly prevalent condition; the incorporation of nutritional guidelines is inadequate, and low-value care is common (4). Cataracts are one of the leading causes of visual impairment and blindness in the elderly. Niazi et al. observed in a meta-analysis of 16 prospective cohort studies conducted in developed countries that every 5 kg/m^2^ increase in body mass index was associated with a 6 and 27% increased risk of age-related cataracts and posterior subscapular cataracts in adults, respectively. Interestingly, Ma et al. observed from 10 waves of the Chinese Longitudinal Healthy Longevity Survey for 16,954 individuals aged 65 and above that drinking tea almost daily was protective against disability, shedding light on good nutritional practice.

The COVID-19 pandemic has significantly affected human society, exacerbating difficult life conditions among vulnerable groups and worsening their adherence to healthy lifestyles, food security, and choices. Dietary changes during COVID-19 have enhanced the burden of malnutrition, with children and older people in low-income households being the most affected, promoting infection, disease progression, and potential death (5). Approximately 30% of children and 60% of adults worldwide are vitamin D deficient and insufficient, respectively (6). Parra-Ortega et al. observed 171 Mexican patients, aged 9–14 years, with chronic kidney disease, decreased serum vitamin D levels, and increased deficiency frequency during the COVID-19 pandemic. Pechdin and Bunditsakulchai also studied that the increased level of malnutrition due to COVID-19 presents an imminent challenge to the government in addressing effective policies, strategies, and interventions. Mattei et al. conducted a double-blind randomized community-based pilot trial to assess the improvement in dietary quality and behaviors through the adaptation and implementation of the Latinos United for Cultural Health Alimentation. The recommendations obtained from this trial include that deep structural messages, in line with evidence-based behavioral theory, should be incorporated into nutrition programs. This study, which was conducted using an electronic technique for data collection during the COVID-19 period, warrants a specific introduction.

In summary, as featured in this Research Topic and available literature, malnutrition remains highly prevalent among vulnerable people. To reduce malnutrition and related health outcomes, increasing food security and accessibility for vulnerable people is essential in both developing and developed countries. Preparedness is of high priority, particularly during pandemics and social crises. The promotion and implementation of educational programs on healthy eating behaviors among children should be strengthened, and dietary quality among adults needs to be improved. Additionally, electronic techniques are recommended for research and promotion of healthy eating behaviors in communities. The suggestions for nutritional interventions and practice programs demonstrated in these articles are supportive.
